# The Ubiquitin Proteasome System Plays a Role in Venezuelan Equine Encephalitis Virus Infection

**DOI:** 10.1371/journal.pone.0124792

**Published:** 2015-04-30

**Authors:** Moushimi Amaya, Forrest Keck, Michael Lindquist, Kelsey Voss, Lauren Scavone, Kylene Kehn-Hall, Brian Roberts, Charles Bailey, Connie Schmaljohn, Aarthi Narayanan

**Affiliations:** 1 National Center for Biodefense and Infectious Diseases, School of Systems Biology, George Mason University, Manassas, United States of America; 2 United States Army Medical Research Institute of Infectious Diseases, Fort Detrick, Maryland, United States of America; 3 Leidos Health Life Sciences, Frederick, Maryland, United States of America; The University of Melbourne, AUSTRALIA

## Abstract

Many viruses have been implicated in utilizing or modulating the Ubiquitin Proteasome System (UPS) to enhance viral multiplication and/or to sustain a persistent infection. The mosquito-borne Venezuelan equine encephalitis virus (VEEV) belongs to the *Togaviridae* family and is an important biodefense pathogen and select agent. There are currently no approved vaccines or therapies for VEEV infections; therefore, it is imperative to identify novel targets for therapeutic development. We hypothesized that a functional UPS is required for efficient VEEV multiplication. We have shown that at non-toxic concentrations Bortezomib, a FDA-approved inhibitor of the proteasome, proved to be a potent inhibitor of VEEV multiplication in the human astrocytoma cell line U87MG. Bortezomib inhibited the virulent Trinidad donkey (TrD) strain and the attenuated TC-83 strain of VEEV. Additional studies with virulent strains of Eastern equine encephalitis virus (EEEV) and Western equine encephalitis virus (WEEV) demonstrated that Bortezomib is a broad spectrum inhibitor of the New World alphaviruses. Time-of-addition assays showed that Bortezomib was an effective inhibitor of viral multiplication even when the drug was introduced many hours post exposure to the virus. Mass spectrometry analyses indicated that the VEEV capsid protein is ubiquitinated in infected cells, which was validated by confocal microscopy and immunoprecipitation assays. Subsequent studies revealed that capsid is ubiquitinated on K48 during early stages of infection which was affected by Bortezomib treatment. This study will aid future investigations in identifying host proteins as potential broad spectrum therapeutic targets for treating alphavirus infections.

## Introduction

The eukaryotic proteasome is a ~2MDa cylindrical shaped protease complex located in the cytoplasm and nucleus and comprises a 20S proteolytic core and one or two 19S regulatory subunits [1,2]. Proteins destined for degradation are tagged with multiple copies of a small 76 amino acid protein, ubiquitin, making the targeted protein identifiable for degradation by the 19S regulatory component [1–3]. The addition of ubiquitin to the targeted proteins is catalyzed by a three step process: an activating enzyme E1, a conjugating enzyme E2, and a ligase E3 [3]. Unfolded polyubiquitin-tagged proteins are fed into the 20S core of the proteasome to be cleaved into smaller peptides [1,2]. The proteasome has three types of catalytic activity: chymotryptic, tryptic, and peptidylglutamyl. The chymotryptic activity cleaves after large hydrophobic residues; cleavage after basic and acidic residues is due to tryptic and peptidylglutamyl activities, respectively [1–3]. The proteasomal inhibitor used in this study, Bortezomib, is a dipeptidyl boronic acid that functions by specifically and reversibly inhibiting the 26S proteasome [1]. Bortezomib is an U.S. Food and Drug Administration (FDA) approved therapeutic proteasome inhibitor (commercially available as Velcade) used to treat multiple myeloma and mantle cell lymphoma [4–6]. The mechanism of inhibition was ascribed to Bortezomib being able to form a tetrahedral adduct with an active threonine site within the proteolytic core; thus making this drug an attractive candidate for drug development [1]. The traditional function of the proteasome was disposing of misfolded proteins or a general recycling role in the cell [1,3,7]. More recently the ubiquitin proteasome system (UPS) has also been described to play a role in non-degradative processes such as cell survival, MHC class I antigen presentation, apoptosis, cell division, NF-κB activation, DNA repair, transcriptional regulation, signal transduction, endocytosis and intracellular trafficking, and chemoresistance [1–3,7].

For several viruses, the UPS has been shown to have antiviral activity. For example, the UPS inhibits entry and post entry steps of both influenza virus [3,7,8] and (mouse) hepatitis virus [3,9]. The replication or expression of human coxsackie 3B virus [10], adenovirus, cytomegalovirus, infectious bursal disease virus, and vesicular stomatitis virus are also inhibited by the UPS [3,11]. In contrast, many viruses have been implicated in utilizing or modulating the UPS to establish a productive infectious cycle [3,7–13]. As proteasomal inhibitors act to deplete free ubiquitin needed to modify viral proteins for efficient viral budding [3], inhibition of the UPS has many effects on viral replication. For example, gag polyprotein processing in HIV infected cells is affected by UPS inhibition, thus decreasing release and infectivity of new virions [12]. In the case of herpes simplex virus (HSV), ubiquitination is required for release of viral DNA such that cytoplasmic DNA sensors recognize the viral DNA and induce an interferon response [13]. In a recent study, inhibition of the host UPS inhibited numerous aspects of the life cycle of different coronaviruses, such as viral entry, RNA synthesis and protein expression [11].

Venezuelan equine encephalitis virus (VEEV) is a New World alphavirus belonging to the *Togaviridae* family [14–18]. VEEV is endemic to South America but has extended to the southern regions of the United States [16,18–20]. VEEV is a mosquito-borne virus that can not only be transmitted to humans by bites from infected mosquitoes but also via an aerosolized route as determined by occurrences of laboratory-acquired infections [17,18,21]. VEEV can easily be weaponized as exhibited in the past [21] and is therefore considered an important biodefense pathogen and select agent. Humans infected with VEEV display symptoms ranging from fever, headache, sore throat, malaise, myalgia, and vomiting to a severe neurological disease and coma [16,18,21,22]. There are currently no FDA-approved vaccines or therapeutics for public use. However, the live-attenuated strain, TC-83, is used as a vaccine for military and at-risk personnel [14,16,18–20,22] and the inactivated trivalent encephalitic virus is more commonly used in horses [23]. VEEV is an enveloped virus that contains a single-stranded positive sense RNA genome (~11,400 nucleotides), which encodes for four nonstructural proteins (nsP1-4) and five structural proteins (capsid, 6K, E1, E2, and E3 envelope glycoproteins) [14,16,22,24,25]. nsP1 is involved in the synthesis and capping of viral RNA [14,26,27]. The newly translated polyprotein is cleaved by the viral proteases nsP2 and nsP4 functions as the RNA polymerase [14,20,26,27]. The role of nsP3 has not yet been fully characterized; however, it has been implicated in RNA synthesis, having a role in pathogenicity in mice [26,27] and interacts with the host inhibitor κB kinase-β (IKKβ) component of the IKK complex [28]. The structural proteins function in the packaging and budding of virion particles from infected cells [24]. The capsid protein originally believed to selectively package only the viral genome is able to cleave itself from the polypeptide [29,30]. Capsid protein has unraveled as a multi-functional viral protein with cytoplasmic and nuclear distribution in infected cells, due to a nuclear localization signal sequence [31]. Capsid functions in host transcriptional shutoff resulting in cytopathogenicity by binding to ribosomes and inhibiting nuclear cytoplasmic trafficking of host proteins [29,31].

In the current study, we hypothesized that the host proteasome system is required for efficient VEEV replication. We have shown that at non-toxic concentrations, Bortezomib was a potent inhibitor of VEEV replication in the astrocyte cell line, U87MG. Bortezomib exhibited broad spectrum inhibitory potential by interfering with the multiplication of virulent strains of VEEV, EEEV and WEEV. Mass spectrometry analysis indicated that capsid protein was ubiquitinated in infected cells which was independently validated by microscopy and immunoprecipitation assays. Subsequent studies suggested that capsid was ubiquitinated on K48 during the early stages of infection which was partly inhibited by Bortezomib. These studies provide evidence for an important role played by the host proteasome in New World alphavirus multiplication and hence identifies the host proteasome as a viable candidate for the development of therapeutics.

## Materials and Methods

### Viruses and Cell Lines

The virulent IAB Trinidad donkey strain (TrD) underwent an initial passage in chick embryos, then 78 passages in guinea pig heart cells to yield TC-78. TC-78 was passaged in chick fibroblast cultures and 12 plaques picked in fetal guinea pig heart cells twice to yield TC-80. One of the plaques was selected for further passage again in fetal guinea pig heart cells (TC-81), which was used for the production of 5 lots of TC-83 vaccine. TC-83 was obtained from BEI Resources. TrD and TC-83 genomes differ at 12 nucleotide positions. Changes in the 5`-noncoding region and the E2 envelope glycoprotein provides attenuation to TC-83 [32]. TC-83 replication has been thoroughly studied *in vitro* and *in vivo* and serves as a biosafety level–2 (BSL-2) model for the fully virulent BSL-3 VEEV TrD strain. Experiments with TC-83 were performed under BSL-2 settings. Experiments with the virulent VEEV TrD were conducted under BSL-3 conditions. A virulent strain of eastern equine encephalitis virus (EEEV) GA97 was obtained from Dr. Jonathan Jacobs (MRIGlobal) and virulent western equine encephalitis virus (WEEV) California 1930 strain was obtained from ATCC. All select agents used are registered with the Centers for Disease Control and Prevention and experiments were conducted at George Mason University’s Biomedical Research Laboratory, which is registered in accordance with Federal select agent regulations. Human astrocytoma cells (U87MG cells) and African green monkey kidney epithelial cells (VERO cells) were maintained in DMEM supplemented with 10% fetal bovine serum, 1% Penicillin/Streptomycin and 1% L-Glutamine at 37°C, 5% CO_2_.

### Inhibitor Studies and Viral Infections

U87MG cells were seeded at 10,000 cells per well in a 96-well plate and the following day were pretreated with inhibitors, Bortezomib (Santa Cruz Biotechnology, sc-217785) or MG132 (Santa Cruz Biotechnology, sc-351846) for 2 hours. The medium containing inhibitor will henceforth be referred to as conditioned medium. The cells were infected for 1 hour to allow for viral adsorption at 37°C. The viral inoculum was removed and replaced with the conditioned media. The cells were incubated at 37°C, 5% CO_2_. The supernatant was collected 24 hours later and stored at -80°C until analyzed.

### Plaque Assay

VERO cells were seeded in a 12-well plate at a density of 1.5x10^5^ cells per well. Viral supernatants were ten-fold serially diluted in DMEM and used to infect VERO cells in duplicate. The plates were incubated for 1 hour at 37°C, 5% CO_2_ with occasional rocking. A 1mL overlay comprising 2X E-MEM and 0.6% agarose (1:1) was added to each of the wells. Once solidified the plates were incubated for an additional 48 hours at 37°C, 5% CO_2_. A 10% formaldehyde solution was then added to the surface of the agarose plugs and incubated for 1 hour at room temperature. The plates were rinsed with diH_2_O and the agarose plugs removed. A 1% crystal violet solution containing 20% ethanol was added to each of the wells and incubated for 30 minutes at room temperature whilst rocking. The plates were rinsed with diH2O, visible plaques counted and the viral titers determined as PFU/mL.

### Cell Viability Assays

Cell viability was measured using a Cell-Titer-Glo luminescent cell viability kit (Promega, G7570) following the manufacturer’s instructions. Briefly, U87MG cells were seeded in 96-well white wall plates at 10,000 cells per well. The next day the cells were treated with varying concentrations of Bortezomib or MG132 and incubated for an additional 24 hours. Cell viability was determined by Cell-Titer-Glo Assay whereby the reagent was added to the cells in a ratio of 1:1. The plate was shaken for 2 minutes and incubated for 10 minutes at room temperature. Luminescence was detected using the DTX 880 multimode detector (Beckman Coulter).

### Time of Addition Study

U87MG cells were seeded in a 96-well plate at a density of 10,000 cells per well and incubated for 24 hours. The conditioned media were removed and cells were infected with TC-83 for 1 hour. The conditioned media were replaced. Infected cells were treated with Bortezomib (0.1μM) or MG132 (1μM) at varying hours post infection. As a control infected cells were treated with DMSO. The supernatants were collected 24 hours post infection and stored at -80°C until ready to perform plaque assays.

### Plasmids, Transfections and Protein Extracts

An ubiquitin plasmid with a HA tag (HA-Ub) was a kind gift from Dr. Benhur Lee (David Geffen School of Medicine, University of California, Los Angeles). Transfections were performed using Attractene Transfection Reagent (Qiagen, 301005) as per manufacturer’s instructions. As a control plasmid pUC19 was used. Transfections were performed for 24 to 48 hours for optimized protein expression. Preparation of whole cell lysates has been previously described [28].

### Immunoprecipitation

U87MG cells were infected with TC-83 at an MOI of 5 and maintained at 37°C, 5% CO_2_. At 2 or 6 hours post infection, cells were collected and lysed in a buffer containing Tris-HCl (pH 7.5), NaCl (120 mM), EDTA (5 mM), NP-40 (0.5%), NaF (50 mM), Na3VO4 (0.2 mM), DTT (1 mM) and one tablet complete protease inhibitor cocktail per 50 ml. Cell lysis was performed on ice for 10 minutes, after which the lysates were centrifuged at 4°C for 10 minutes at 10,000 rpm. Supernatants were transferred to new tubes and protein quantitated by Bradford protein assay (BioRad, Hercules, CA, USA). Two milligrams of total protein was incubated overnight with rotation, at 4°C with VEEV capsid antibody (BEI Resources, NR 9403) or an isotype IgG control antibody. A 30% slurry of Protein A+G beads (Calbiochem, Rockland, MA) was added to the immunoprecipitates (IPs) and incubated for 2 hours with rotation at 4°C. The IPs were centrifuged briefly and beads were washed once with TNE300 + 0.1% NP-40, TNE150 + 0.1% NP-40 and TNE50 + 0.1% NP-40.

### Western Blot Analysis

Whole cell lysates were separated on a 4–20% Tris-Glycine Gel and transferred to a polyvinyl difluoride (PVDF) membrane using the iBlot gel transfer system (Invitrogen). Primary antibodies to VEEV Capsid, Ubiquitin (Abcam, ab7780), and Anti-Ubiquitin (linkage-specific K48) antibody (Abcam, ab140601) were used according to the manufacturer’s instructions. The blots were incubated with respective secondary HRP-coupled antibody. The membranes were visualized by chemiluminescence using SuperSignal West Femto Maximum Sensitivity Substrate Kit (ThermoScientific) and a BIO-RAD Molecular Imager ChemiDoc XRS system (BIO-RAD).

### Liquid Chromatography tandem Mass Spectrometry (LC-MS/MS)

U87MG cells were either transfected with pUC19 or HA-Ub for 24 hours. Transfected cells were infected with TC-83 at an MOI of 5. Infected cells were maintained at 37°C, 5% CO_2_. At 6 hours post infection cells were collected, lysed and processed as described above. Two milligrams of total protein, quantified by Bradford assay was incubated overnight with rotation, at 4°C with normal mouse IgG antibody (Santa Cruz Biotechnology, sc-2025) or α-HA antibody (Santa Cruz Biotechnology, sc-7392). A 30% slurry of Protein A+G beads (Calbiochem, Rockland, MA) was added to the IPs and incubated for 2 hours with rotation at 4°C. The IPs were centrifuged briefly and beads were washed twice with TNE300 + 0.1% NP-40, followed by a single wash with TNE150 + 0.1% NP-40.

LC-MS/MS analysis was carried out as previously described [28,33]. Briefly, samples were first lysed in 8M urea, after which, they were reduced using DTT and acetylated using iodoacetamide. The reduced and alkylated proteins were trypsin digested (Trypsin, Promega) for 4 hours at 37°C. The digested peptides were eluted using ZipTip purification (Millipore) and analysis of the peptides was performed by LTQ-tandem MS/MS equipped with a reverse-phase liquid chromatography nanospray (ThermoFisher). After sample injection, the column was washed for 5 minutes at 200 nl/min with 0.1% formic acid; peptides were eluted using a 50-minute linear gradient from 0 to 40% acetonitrile and an additional step of 80% acetonitrile (all in 0.1% formic acid) for 5 minutes. The LTQ-MS was operated in a data-dependent mode in which each full MS scan was followed by five MS-MS scans where the five most abundant molecular ions were dynamically selected and fragmented by collision-induced dissociation using normalized collision energy of 35%. Tandem mass spectra were matched against the National Center for Biotechnology Information human database by Sequest Bioworks software (ThermoFisher) using full tryptic cleavage constraints and static cysteine alkylation by iodoacetamide.

### Fluorescence In Situ Hybridization (FISH)

The VEEV TC-83 genome sequence was obtained from GenBank (L01443.1). The first 8000 bases of the genome were used to design RNA FISH probes using Stellaris RNA FISH Probe Designer Version 4.0 (Biosearch Technologies). A probe set of 48 individual probes was generated and each probe was tagged with a Quasar 570 fluorophore. In order to detect viral RNA, U87MG cells were seeded onto #1.0 coverslips in 24-well plates at a density of 54,000 cells per well. Cells were pre-treated with 0.1μM Bortezomib or DMSO for 2 hours. The conditioned media were removed and the treated cells were infected with TC-83 at an MOI of 5. After 1 hour incubation, the viral inoculum was replaced with the conditioned media. At 0, 4 or 8 hours post infection, cells were fixed in 3.7% formaldehyde for 10 minutes, washed, and placed in 70% ethanol overnight at 4°C. Samples were then processed for RNA FISH using the Stellaris TC-83 viral RNA probes according to the manufacturer’s protocol. For the hybridization step, TC-83 viral RNA probes were diluted to a concentration of 250 nM in formamide hybridization buffer and 100μL of probe solution was added to the center of each coverslip. Following the RNA hybridization steps, cells were washed with a PBS solution containing DAPI. The DAPI solution was washed off cells and replaced with PBS. Coverslips were mounted onto slides using Vectashield hardmount mounting medium and samples were imaged using a Zeiss 700 confocal microscope. All images were obtained using a 20X or 63X objective. Vero cells were infected with RVFV (strain MP-12) at an MOI of 0.3 for 24 hours. Following infection, cells were processed for RNA FISH as described above. Cells were then stained with an anti-nucleocapsid antibody (R3-1D8-1).

### Immunofluorescence

U87MG cells were seeded at a density of 20,000 cells per well in an 8-well chambered slide. The cells were either Bortezomib treated or DMSO treated, uninfected (Mock) or infected as described above. Cells were fixed with 4% paraformaldehyde, permeabilized with 0.1% Triton X-100 in 1X PBS, followed by blocking with 1% BSA/ 0.3M glycine/0.025% Triton X-100, in PBS for 30 minutes. The slides were incubated with primary antibody for 1 hour in the dark at room temperature. The slides were then washed three times with PBS and incubated with respective secondary antibody Alexa Fluor antibodies (Invitrogen) for 1 hour in the dark at room temperature. Slides were washed three times with PBS and incubated with DAPI for 10 minutes in the dark at room temperature. Following an additional PBS wash, the slides were mounted with Fluoromount G (SouthernBiotech, 0100–01) and stored in the dark, at 4°C overnight. The cells were imaged using a Nikon Eclipse TE2000-U. Images were taken with a 60X objective. Co-localization was confirmed with Z-stacks taken at 0.1μm increments.

### Quantitative RT-PCR (q-RT-PCR)

U87MG cells were pre-treated with 0.1μM Bortezomib or DMSO for 2 hours and then infected with TC-83 at an MOI: 0.1. At 0, 2, 4, 6, and 8 hours post infection cells were lysed using the MagMAX-96 Total RNA Isolation Kit (Life Technologies, AM1830) as per the manufacturer’s instructions. As additional controls, cells were either pre-treated with 0.1μM Bortezomib or DMSO for 2 hours, then infected with TC-83 (MOI: 0.1) and the cells lysed at 24 hours post infection. Viral RNA was quantitated using q-RT-PCR with primers and probe for nucleotides 7931–8005 of VEEV TC-83 [34]. q-RT-PCR cycling conditions were as follows: 1 cycle at 50°C for 30 minutes, 1 cycle at 95°C for 2 minutes and 40 cycles at 95°C for 15 seconds and 61°C for 1 minute using the StepOne Plus Real Time PCR system [34]. The primer and probe pairs used were originally described by Julander; forward primer (TCTGACAAGACGTTCCCAATCA) and reverse primer (GAATAACTTCCCTCCGACCACA) and Taqman probe (5’ 6-carboxyfluorescein-TGTTGGAAGGGAAGATAAACGGCTACGC-6-carboxy- *N*,*N*,*N*′,*N*′-tetramethylrhodamine-3′) [35]. q-RT-PCR assays were performed using BioRad iTaq Universal Probes one-step 2x mix (BioRad, 172–5140). The absolute quantification was calculated based on the threshold cycle (Ct) relative to the standard curve.

### Viral purification by sucrose density gradient centrifugation

Pooled supernatants from VEEV infected VERO cells were collected 48 hours post infection and centrifuged at 10,000 RPM for 30 minutes. Five percent polyethylene glycol (6000) and 2.3% sodium chloride was added to the supernatant and allowed to stir overnight at 4°C. The stirred supernatant was centrifuged at 10,000 RPM for 30 minutes. The supernatant was discarded and the pellet was resuspended in PBS. The virus suspension was layered on top of a freshly prepared 20%-60% continuous sucrose density gradient. Gradients were centrifuged at 36,000 RPM at 4°C overnight. Three millimeter fractions were taken from the top down and resuspended in a buffer comprising 10mM Tris (pH 8.0), 2mM MgCl_2_ and 4% sucrose. Fractions were titered by plaque assay to determine the fraction that had the highest purity and infectivity for downstream immunoprecipitation assays.

### Statistical Analysis

Individual experiments were performed in triplicate. RNA FISH experiment was performed as 2 independent experiments in duplicate. Unless otherwise stated, graphs are the average of 2 independent experiments. All experimental results were expressed as the arithmetic mean. Standard deviations were calculated from 2 independent experiments and are represented thusly. All statistical analyses was performed with the unpaired, two-tailed Student T-test using GraphPad’s—QuickCalcs software, (GraphPad).

## Results

### Bortezomib inhibits TC-83 multiplication in U87MG cells

**Fig 1:**
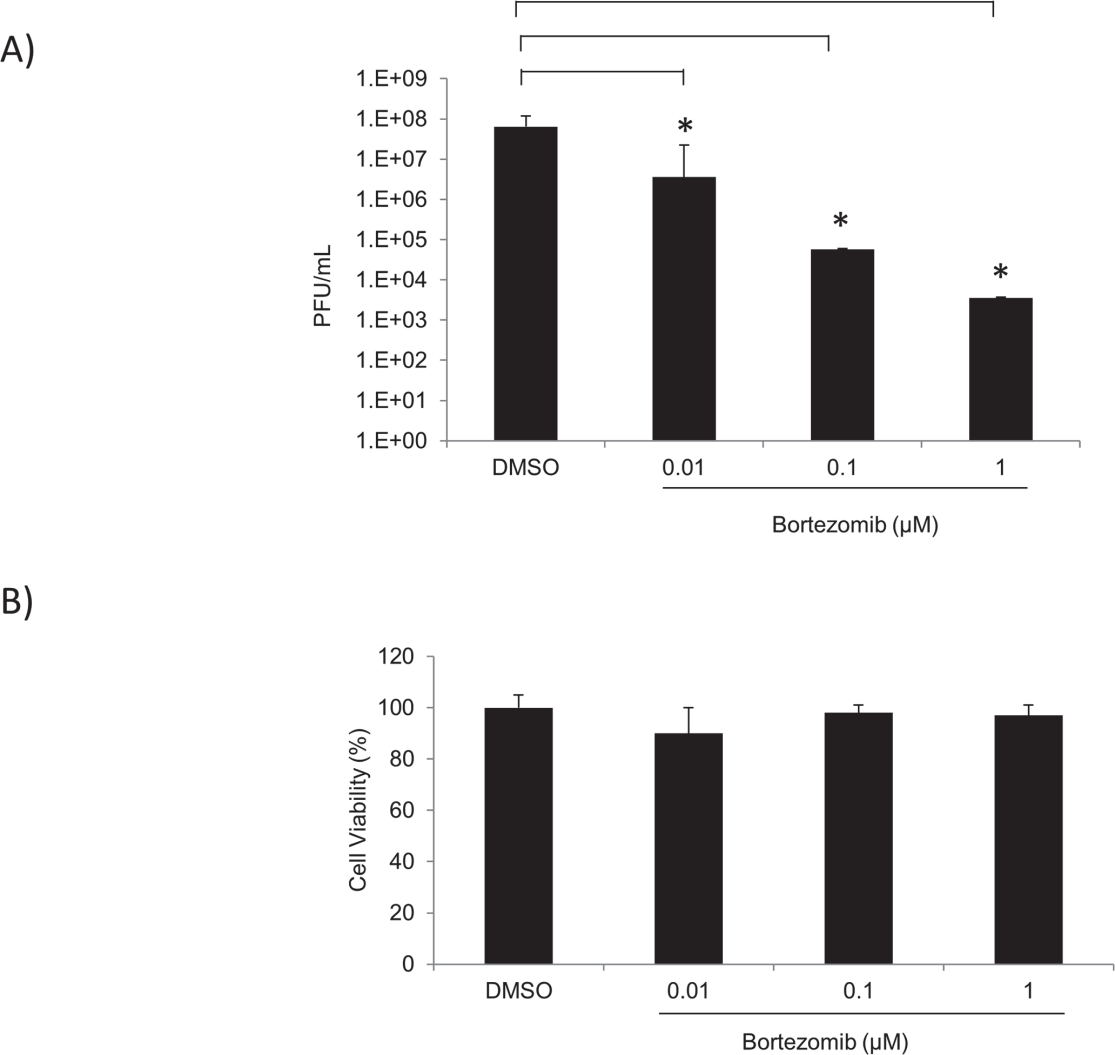
Bortezomib inhibits TC-83 multiplication in U87MG cells. A) U87MG cells were seeded in a 96-well plate at a density of 10,000 cells per well. Cells were either pre-treated with DMSO or 0.01μM, 0.1μM, or 1μM of Bortezomib in triplicate for 2 hours. The conditioned media were removed prior to infection with TC-83 (MOI: 0.1) for 1 hour. The viral inoculum was removed and replaced with conditioned media. Supernatants were collected 24 hours post infection and stored at -80°C until analyzed by plaque assays to determine infectious viral titers as PFU/mL. B) U87MG cells were seeded in a white walled 96-well plate at a density of 10,000 cells per well. Cells were either pre-treated with DMSO or 0.01μM, 0.1μM, or 1μM of Bortezomib in triplicate for 24 hours. Cell viability was measured using a Cell-Titer-Glo luminescent cell viability kit whereby the reagent was added to the cells in a 1:1 ratio. The plate was shaken for 2 minutes and incubated for 10 minutes at room temperature. Luminescence was detected using the DTX 880 multimode detector (Beckman Coulter). The arithmetic means are illustrated graphically. The graphs are representative of 2 independent experiments performed in triplicate. Standard deviations were calculated from 2 independent experiments. p<0.05(*).

**Fig 2:**
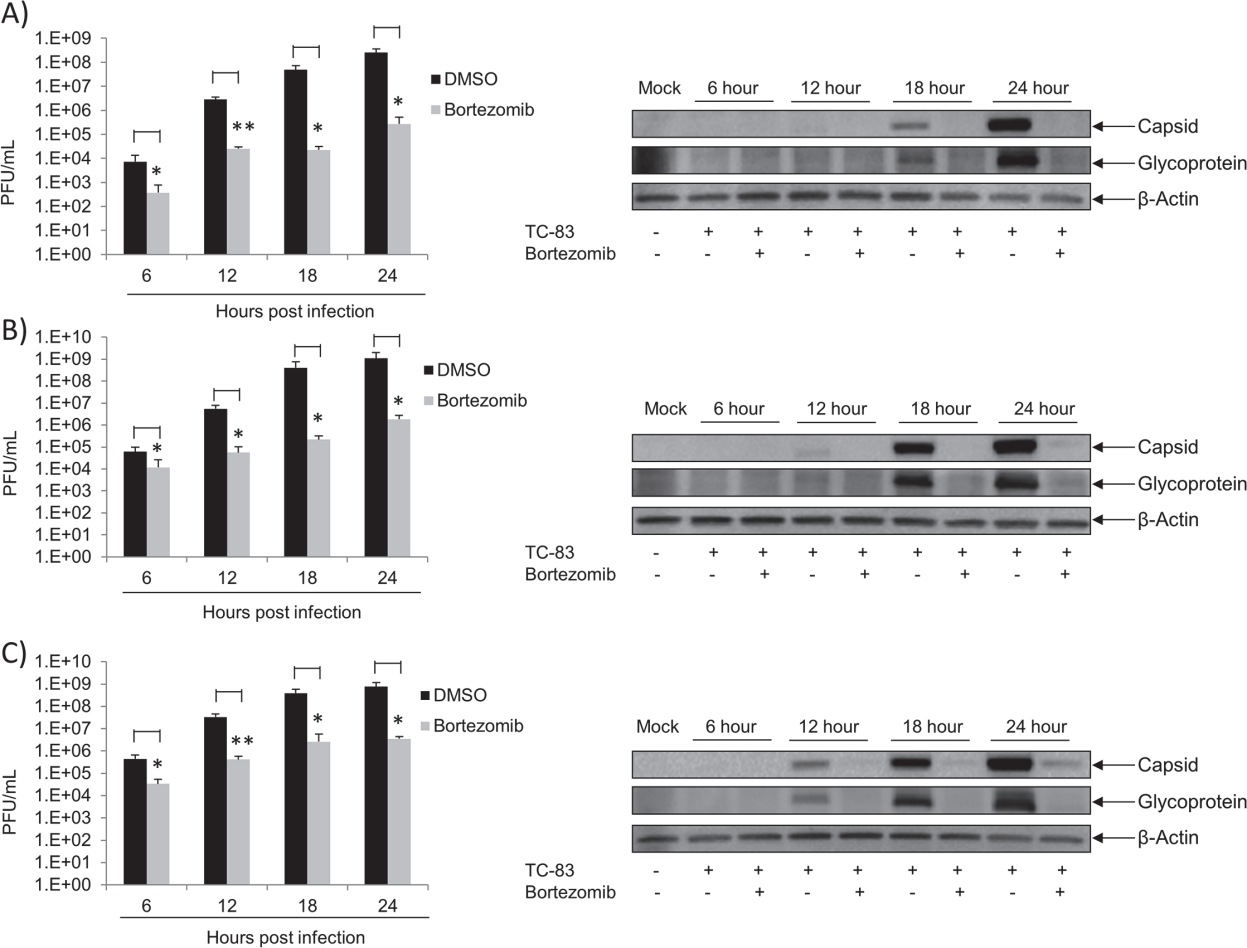
Bortezomib inhibition is independent of viral load. U87MG cells were seeded in a 96-well plate at a density of 10,000 cells per well. Cells were pre-treated with Bortezomib (0.1μM) or DMSO in triplicate for 2 hours. Treated cells were infected with TC-83 at MOI: 0.1 (A) MOI: 1 (B) or MOI: 5 (C). The viral inoculum was removed and replaced with conditioned media. At 6, 12, 18 and 24 hours post infection supernatants were collected and analyzed by plaque assay. The graph is representative of 2 independent experiments performed in triplicate. Standard deviations were calculated accordingly. p<0.05(*). p≤0.0001 (**). U87MG cells were seeded in a 12-well plate at a density of 100,000 cells per well. Cells were pre-treated with Bortezomib (0.1μM) or DMSO for 2 hours. Treated cells were infected with TC-83 at MOIs (0.1, 1 or 5) (right panels of A, B and C respectively). The viral inoculum was removed and replaced with conditioned media. At 6, 12, 18 and 24 hours post infection cell lysates were collected and analyzed by western blot. The images are representative of 2 independent experiments.

The UPS pathway is of great importance in the regulation of cellular processes. Therefore, many viruses take advantage of this system in order to enhance viral replication [3,4,9,11]. To determine if the FDA approved drug, Bortezomib (a proteasome inhibitor) inhibited TC-83 multiplication, U87MG cells were pre-treated with 0.01μM, 0.1μM or 1μM Bortezomib for 2 hours. Treated cells were then infected with TC-83 for 1 hour, after which the conditioned media were replaced. Supernatants were collected 24 hours post infection and stored at -80°C until analyzed by plaque assay. Cells treated with DMSO served as controls. Fig 1A illustrated that in the presence of Bortezomib, TC-83 multiplication decreased in a dose-dependent fashion when compared to the DMSO control. Bortezomib decreased TC-83 multiplication by ~2 logs at 0.01μM and by 3 logs at 0.1μM. At 1μM Bortezomib a 4-log decrease was observed (Fig 1A). To ensure that the inhibition was not due to the toxicity of the drug, U87MG cells were pre-treated with DMSO or the same concentrations of Bortezomib as in Fig 1A. Cell viability was determined by Cell-Titer-Glo Assay 24 hours post treatment. In Fig 1B Bortezomib was found to not be toxic to U87MG cells at the concentrations tested. These results indicated that Bortezomib inhibits TC-83 multiplication in a dose-dependent manner in U87MG cells at nontoxic concentrations. Subsequent experiments were performed with 0.1μM Bortezomib, as at this concentration TC-83 multiplication was inhibited with no toxicity to the cells. To determine if the observed inhibition was independent of multiplicity of infection (MOI), Bortezomib treated U87MG cells were infected at either MOI: 0.1, 1 or 5. At 6 hour intervals from 0 to 24 hours post infection, supernatants were collected and analyzed by plaque assay for infectious viral titers. Fig 2 (left panels) demonstrated that Bortezomib inhibited TC-83 multiplication at all MOIs tested when compared to the DMSO controls. Western blot analysis of infected lysates illustrated an increase in viral structural protein expression (capsid and glycoprotein) with an increase in viral load. In the presence of Bortezomib however, a decrease in viral structural proteins was observed when compared to the DMSO controls (Fig 2 right panels). The observed decrease in capsid and glycoprotein expression was not affected by the increase in MOI. Taken together these results indicated that at the non-toxic concentration, Bortezomib inhibited TC-83 multiplication independently of the initial viral load.

### Bortezomib inhibits TC-83 multiplication at early stages of infection in U87MG cells

**Fig 3:**
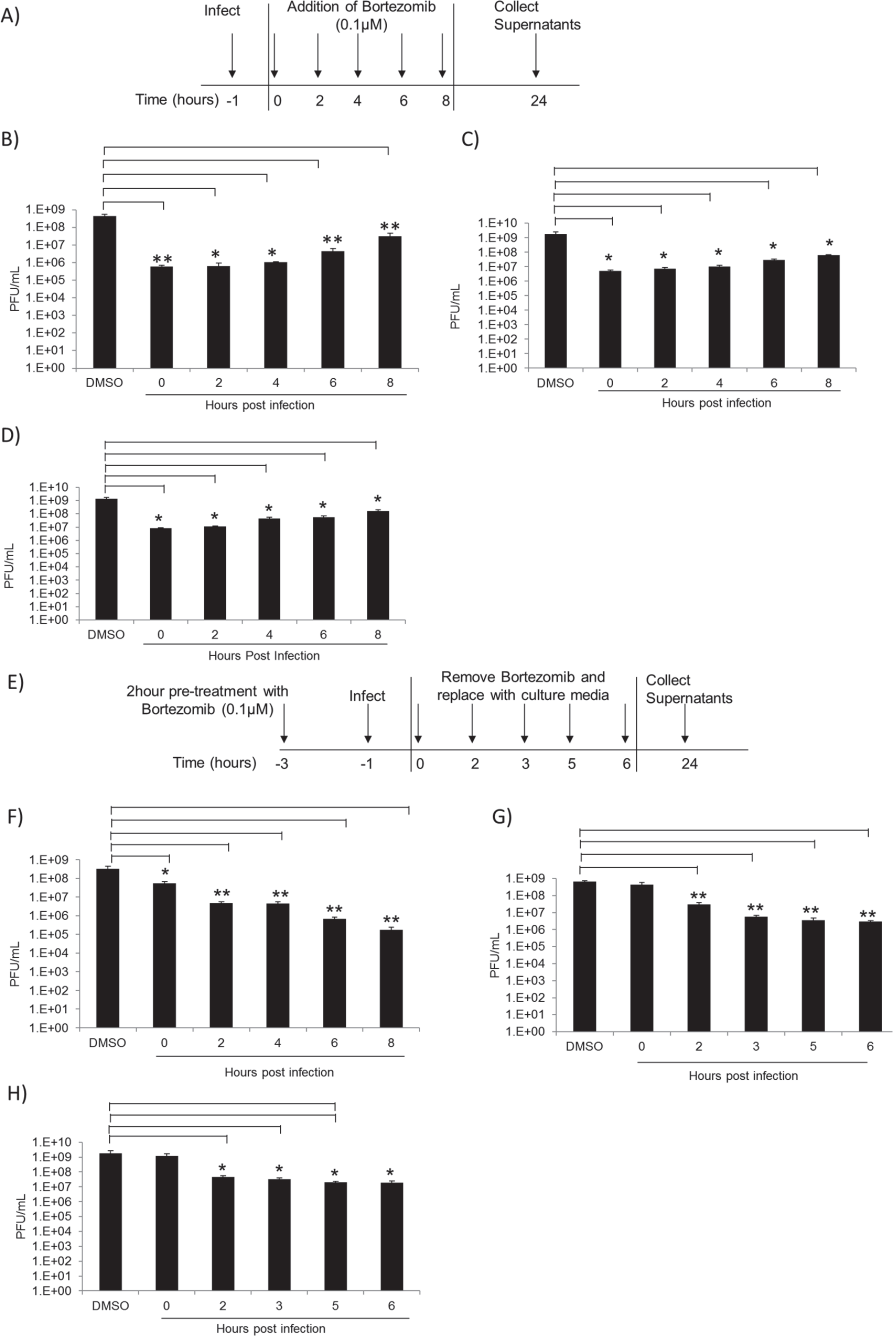
Bortezomib inhibits TC-83 multiplication at early stages of infection. A) Representative schematic of the time of addition assay. U87MG cells were seeded in a 96-well plate at a density of 10,000 cells per well. Cells were infected in triplicate with TC-83 at MOI: 0.1 (B), MOI: 1 (C) or MOI: 5 (D) for 1 hour. Infected cells were treated with 0.1uM of Bortezomib at 2 hour intervals from 0 hours post infection to 8 hours post infection. As controls, infected cells were treated with DMSO. Supernatants were collected 24 hours post infection and analyzed by plaque assay for infectious viral particles. E) Representative schematic of the modified time of addition assay. U87MG cells were pre-treated with Bortezomib and then infected with TC-83 at MOI: 0.1 (F), MOI: 1 (G) or MOI: 5 (H) for 1 hour. The viral inoculum was removed and replaced with conditioned media. At 0, 2, 3, 5, and 6 hours post infection the conditioned media were removed and replaced with culture media. As controls infected cells were treated with DMSO. At 24 hours post infection supernatants were collected and analyzed by plaque assay. The graph is representative of 2 independent experiments. Standard deviations were calculated accordingly. p<0.05(*). p≤0.0001 (**).

Our initial experiment to evaluate efficacy of Bortezomib in inhibiting TC-83 multiplication involved pretreating the cells with the inhibitor. To determine whether Bortezomib could function as an effective inhibitor of viral multiplication if introduced post infectious exposure, a time of addition assay was performed. The objective of this experiment was to determine the time post exposure when Bortezomib treatment was still efficacious in inhibiting the virus. The schematic for this assay is represented in Fig 3A. Briefly, U87MG cells were infected with TC-83 at MOI: 0.1 (B), MOI: 1 (C) or MOI: 5 (D) for 1 hour and Bortezomib was added to the infected cells at 2 hour intervals from 0 to 8 hours post infection. Infected cells treated with DMSO served as controls. Supernatants were collected 24 hours post infection and analyzed by plaque assay for infectious viral particles. In Fig 3B–3D, a significant decrease in TC-83 multiplication was observed in Bortezomib treated cells at all time points investigated when compared to the DMSO control, despite the increase in viral load. These observations indicated that Bortezomib can be successfully applied to inhibit viral multiplication if added up to 8 hours post infection.

We next hypothesized that if the drug was provided post exposure, the longer the drug was available, the greater would be the extent of inhibition. This also supports the well-known notion that Bortezomib mediated inhibition was reversible and, if Bortezomib was removed from the system, viral multiplication would resume. The schematic for this modified time of addition assay is represented by Fig 3E. U87MG cells were subjected to a 2 hour pre-treatment with Bortezomib followed by infection with TC-83 at MOI: 0.1 (F), MOI: 1 (G) or MOI: 5 (H). After a 1 hour adsorption, the conditioned media were added back to the infected cells. At the time points indicated, the conditioned media were replaced with culture media. All supernatants were collected 24 hours post infection and analyzed by plaque assay. Fig 3F–3H indicated that at the earlier time points of Bortezomib removal, TC-83 multiplication resumed; whereas at the later time points of Bortezomib removal a decrease in viral titers was observed. Furthermore, the observed decrease was not influenced by the increase in viral titers. This data suggests that there is greater inhibition of TC-83 multiplication with prolonged Bortezomib treatment.

### Other proteasome inhibitors inhibit TC-83 multiplication

**Fig 4:**
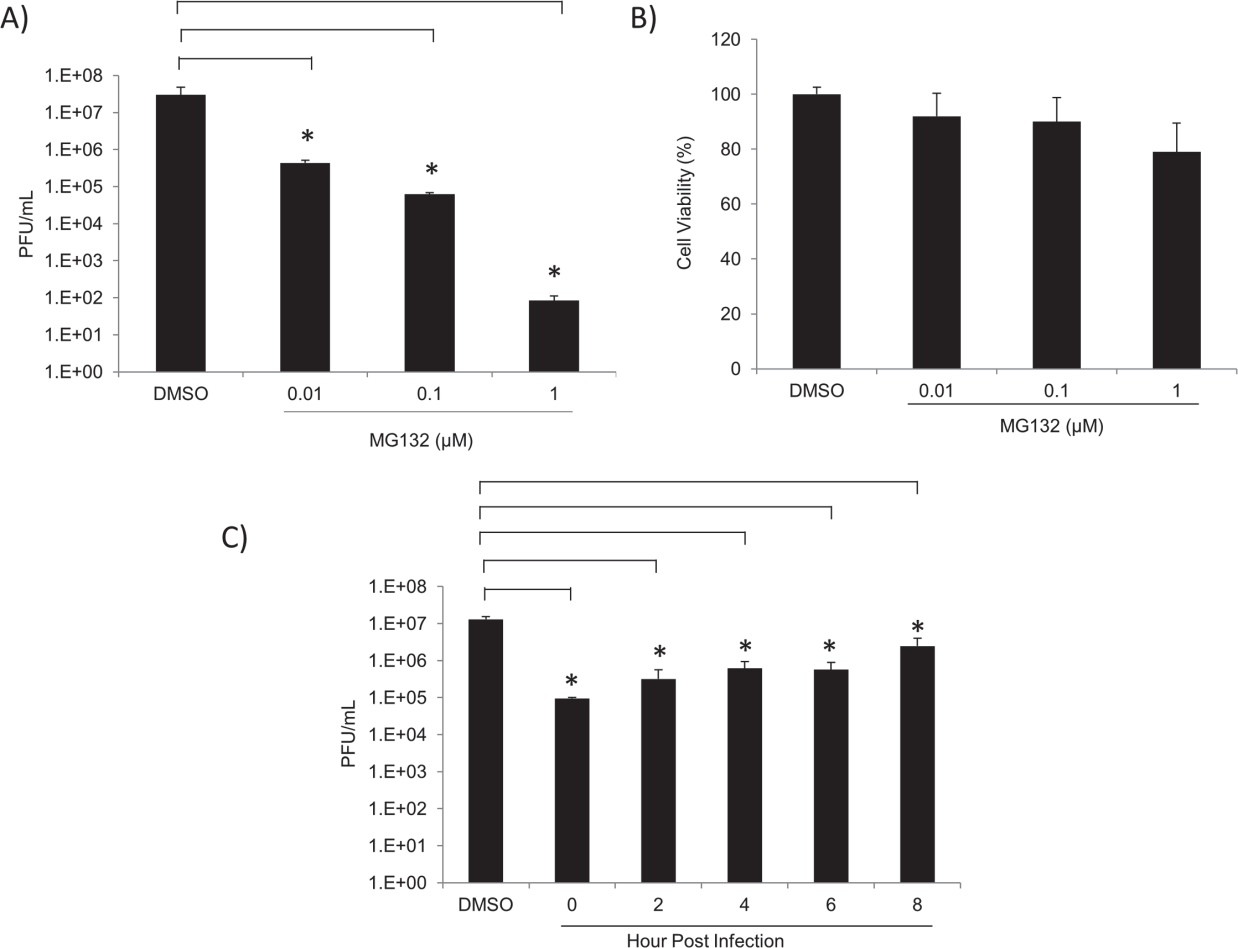
Other proteasomal inhibitors decrease TC-83 multiplication in U87MG cells. A) U87MG cells were seeded in a 96-well plate at a density of 10,000 cells per well. Cells were pre-treated with MG132 at 0.01μM, 0.1μM, or 1μM concentrations for 2 hours. Treated cells were infected with TC-83 at MOI: 0.1 for 1 hour. Supernatants were collected 24 hours post infection and infectious viral titers (PFU/mL) were assessed by plaque assay. B) U87MG cells were seeded in a 96-well plate at a density of 10,000 cells per well. Cells were treated with either DMSO or varying concentrations of MG132. Cell viability was determined 24 hours post-treatment using Cell-Titer-Glo Reagent as per manufacturer’s instructions. The graphs are representative of 3 independent experiments, each performed in triplicate. Standard deviations were calculated from 3 independent experiments and are represented thusly. C) U87MG cells were seeded in a 96-well plate at a density of 10,000 cells per well. Cells were infected in triplicate with TC-83 at MOI: 0.1 for 1 hour. Infected cells were treated with 1μM of MG132 at 2 hour intervals from 0–8 hours post infection. As controls infected cells were treated with DMSO. Supernatants were collected 24 hours post infection and analyzed by plaque assay for infectious viral particles (PFU/mL). The arithmetic means are illustrated graphically. The graph is representative of 2 independent experiments performed in triplicate. Standard deviations were calculated accordingly. p<0.05(*).

To verify that the effects of Bortezomib on TC-83 multiplication were mediated by an impaired UPS; we tested another proteasomal inhibitor, MG132. The peptide aldehyde MG132 inhibits the 20S proteasome activity by binding to the active sites of the beta subunits hence blocking proteolytic activity of the 26S proteasome [36]. To determine if MG132 reduced viral titers similar to the reduction observed with Bortezomib, U87MG cells were pre-treated with 0.01μM, 0.1μM, or 1μM of MG132 for 2 hours. The conditioned medium was removed and the cells were infected with TC-83 for 1 hour. The conditioned media were replaced and the supernatants collected 24 hours post infection. Plaque assays were performed to determine viral titers and are depicted in Fig 4A. As shown in Fig 4A, a 2, 3, and 6-log decrease in viral titers was observed with 0.01μM, 0.1μM, and 1μM MG132, respectively, when compared to the DMSO control. To ensure that the inhibition was not due to the toxicity of the drug, cell viability was determined by Cell-Titer-Glo Assay. MG132 was found not to be toxic to the cells at all concentrations tested (Fig 4B). A time-of-addition assay identical to that in Fig 3B was performed. Fig 4C indicated that MG132 was also capable of inhibiting TC-83 multiplication when provided in a post exposure manner. Taken together, these results suggested that MG132 inhibited TC-83 multiplication in a dose-dependent manner.

### Bortezomib treatment down-regulates virulent new world alphavirus multiplication

**Fig 5:**
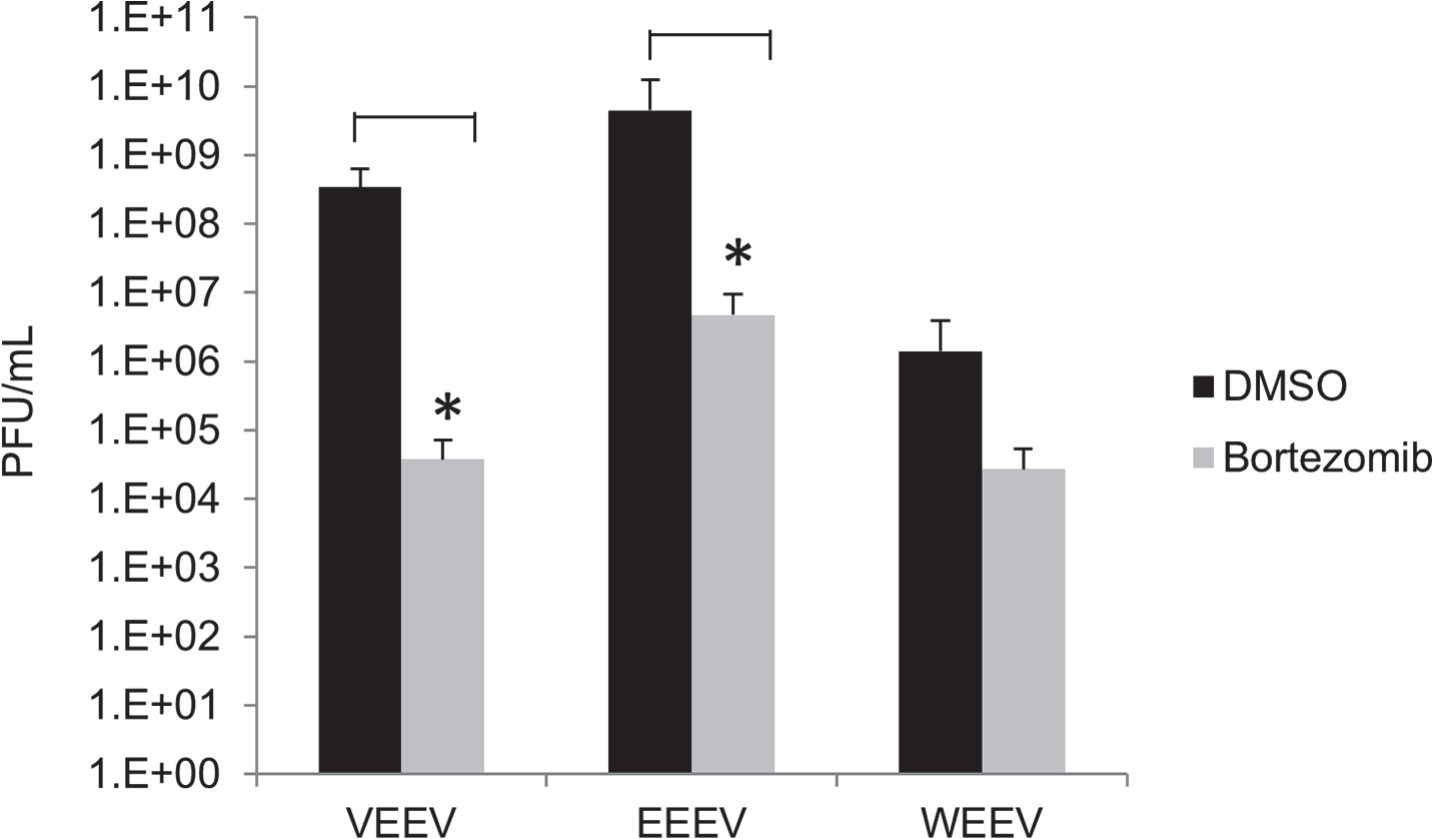
Inhibition of wild type alphaviruses by Bortezomib. U87MG cells were pre-treated with Bortezomib at 0.1μM for 2 hours and then infected with VEEV TrD, EEEV strain GA97, and WEEV California 1930 strain at an MOI: 0.1 for 1 hour. Supernatants were collected 24 hours post infection and analyzed by plaque assay for infectious viral particles (PFU/mL). The arithmetic means are illustrated graphically. The graph is representative of 3 independent experiments where each experiment was performed in triplicate. Standard deviations were calculated accordingly. p<0.05(*).

Thus far our results have shown that Bortezomib effectively decreased TC-83 multiplication by interfering with early stages in the infectious process, which prompted us to investigate if a similar trend could be observed with the virulent strains of all New World alphaviruses. U87MG cells were pre-treated with DMSO or Bortezomib and then infected with VEEV TrD, EEEV strain GA97, or WEEV California 1930 strain for 1 hour. Conditioned media were replaced and the supernatants were collected 24 hours post infection. Plaque assays were performed to determine infectious viral particles. In Fig 5 Bortezomib treatment decreased viral multiplication of TrD by 4 logs, EEEV by 3 logs and WEEV by 2 logs when compared to their respective DMSO controls. These results suggested that Bortezomib is a broad spectrum inhibitor of virulent New World alphaviruses.

### Intracellular viral RNA decreases with Bortezomib treatment

**Fig 6:**
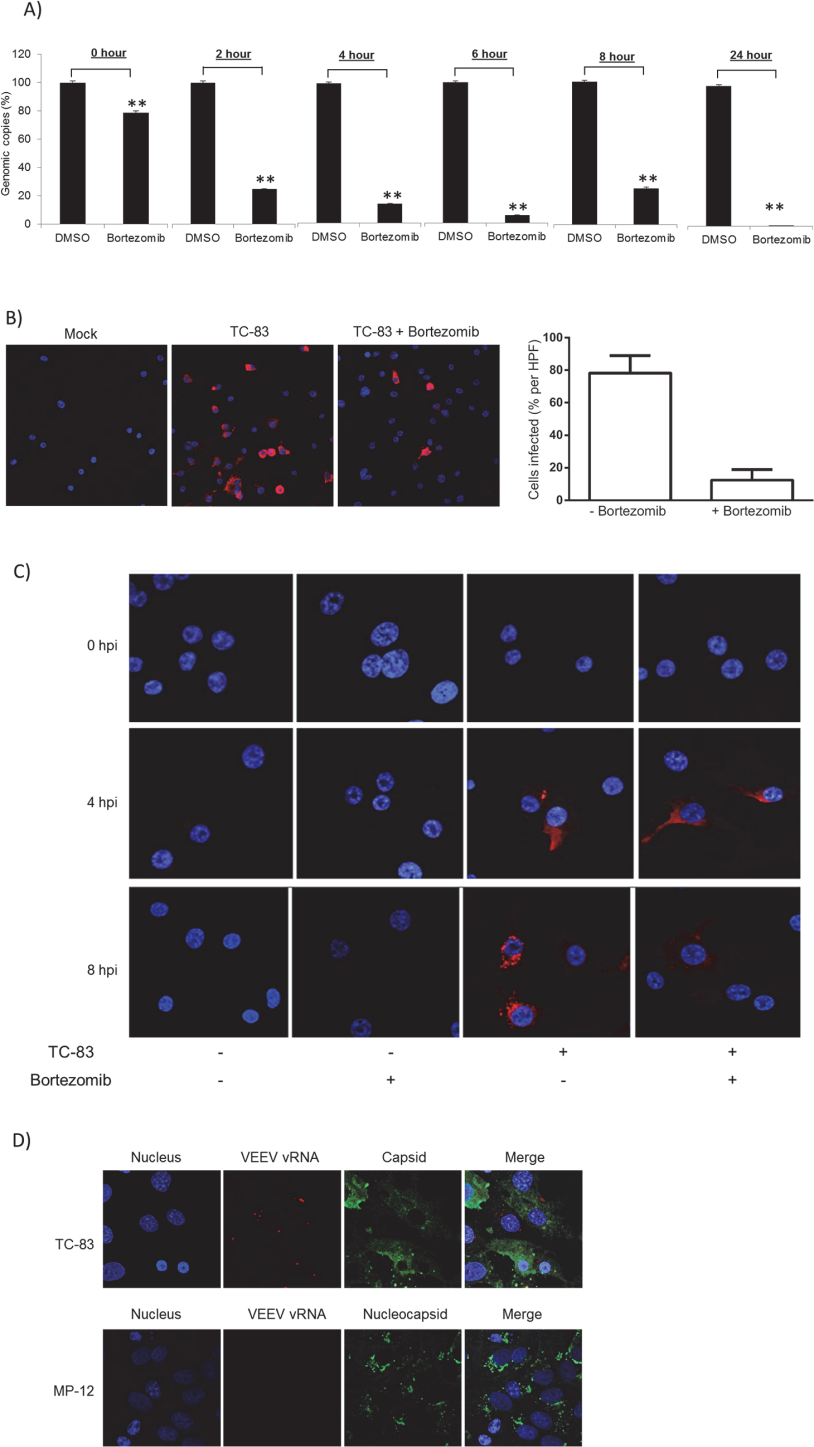
Bortezomib decreases intracellular viral RNA. A) U87MG cells were seeded in a 96-well plate at a density of 10,000 cells per well. Cells were pre-treated with 0.1μM Bortezomib or DMSO for 2 hours and then infected with TC-83 (MOI: 0.1) for 1 hour. At 0, 2, 4, 6, and 8 hours post infection cells were lysed with MagMAX-96 Total RNA Isolation Kit. As controls, cells were either pre-treated with 0.1μM Bortezomib or DMSO for 2 hours, then infected with TC-83 (MOI: 0.1) and the cells lysed at 24 hours post infection. Levels of viral RNA were quantified by q-RT-PCR using VEEV specific primers. The cycling conditions and probe primer pair sequences are described in the methods section. The graphs represent an average of 2 independent experiments where each experiment was performed in triplicate. Standard deviations were calculated accordingly. p≤0.0001 (**). U87MG cells were seeded onto #1.0 coverslips in 24-well plates at a density of 54,000 cells per well. Cells were pretreated with Bortezomib for 2 hours and then infected with TC-83 (MOI: 5) (B and C). B) After 8 hours of infection, the cells were fixed and processed for RNA FISH as described in the materials and methods section. Following FISH staining, cells were imaged using a Zeiss 700 confocal microscope with a 20X objective and at least 10 high-powered fields (HPFs) were obtained for each sample. The percentage of infected to uninfected cells was calculated per HPF for each condition. A representative 20X objective image is shown with viral RNA in red and nuclei in blue. C) After 0, 4 and 8 hours of infection, the cells were fixed and processed for RNA FISH as described in the materials and methods section. Following FISH staining, cells were imaged using a Zeiss 700 confocal microscope. A representative 63X objective image is shown with viral RNA in red and nuclei in blue. D) VERO cells were infected with TC-83 or Rift Valley Fever Virus (RVFV) (strain MP-12) for 24 hours. Cells were then processed for RNA FISH and stained with VEEV vRNA specific FISH probes as described in the materials and methods section. Following the RNA FISH staining, the VEEV infected cells were immunostained with an anti- VEEV capsid antibody and the RVFV infected cells were immunostained with an anti-RVFV nucleocapsid antibody. Nuclei were detected using DAPI. Images are shown as condensed Z-stacks. The images are representative of 2 independent experiments performed in duplicate.

The observed results thus far led us to hypothesize that the mechanism by which Bortezomib may inhibit viral multiplication may involve impaired early events, including viral capsid stabilization which may interfere with release of viral genomic material to enable subsequent RNA replication and viral protein synthesis. Plaque assays performed using DMSO-treated or Bortezomib-treated cells revealed less infectious virus to be present in the supernatant of drug-treated cells (Figs 2 and 3). To address our hypothesis that Bortezomib treatment may interfere with the extent of viral RNA replication, we quantified the amount of intracellular viral RNA during the early stages of the infectious process in the cytoplasm of drug-treated cells by q-RT-PCR using VEEV specific primers. Pre-treated infected cells were lysed at various time points post infection for analysis of intracellular viral RNA. Fig 6A illustrates a decrease in VEEV genomic copies in Bortezomib treated cells when compared to the corresponding DMSO controls. In addition, we utilized RNA FISH with probes designed specifically to detect intracellular VEEV genomic RNA in order to determine if there are changes in the intracellular distribution pattern of the RNA in the presence of Bortezomib (Fig 6B and 6C). FISH analysis illustrated that at 8 hours post infection Bortezomib treatment inhibited viral RNA production when compared to the control. Following FISH staining, cells were imaged using a 20X objective and at least 10 high-powered fields (HPFs) were obtained for each sample. The percentage of infected to uninfected cells was calculated per HPF for each condition. An approximately 60% decrease in viral RNA production (as determined by fluorescent signal) was observed with Bortezomib treatment. At 0, 4 and 8 hours post infection a progressive increase in intracellular viral RNA in the DMSO-treated cells was observed, which ultimately reorganized into tight cytoplasmic punctate foci-like regions at later time points. The VEEV-specific FISH probes did not detect viral RNA at 0 hours post infection (Fig 6C). Such a robust RNA replication and reorganization was less frequent in Bortezomib-treated cells supporting the possibility that the observed decrease in extracellular infectious virions may be attributed to a potential decrease in intracellular viral replication and RNA organization into relevant cytoplasmic structures. To ensure the specificity of the VEEV RNA FISH probes, we utilized Rift Valley Fever Virus (strain MP-12). Twenty four hours post infection with TC-83 or MP-12, VERO cells were fixed and processed for RNA FISH stained with VEEV vRNA specific FISH probes (red) (Fig 6D). Subsequent to the FISH staining, TC-83 and MP-12 infected cells were immunostained to detect levels of infection. We did not observe any co-localization of VEEV capsid with viral RNA in the TC-83 infected cells. Thus, the VEEV RNA FISH probes were specific to VEEV and it did not interact with MP-12 RNA. In summary, our data reveals that Bortezomib treatment resulted in decreased intracellular viral RNA levels and altered RNA distribution in infected cells.

### Ubiquitination of TC-83 capsid

**Fig 7:**
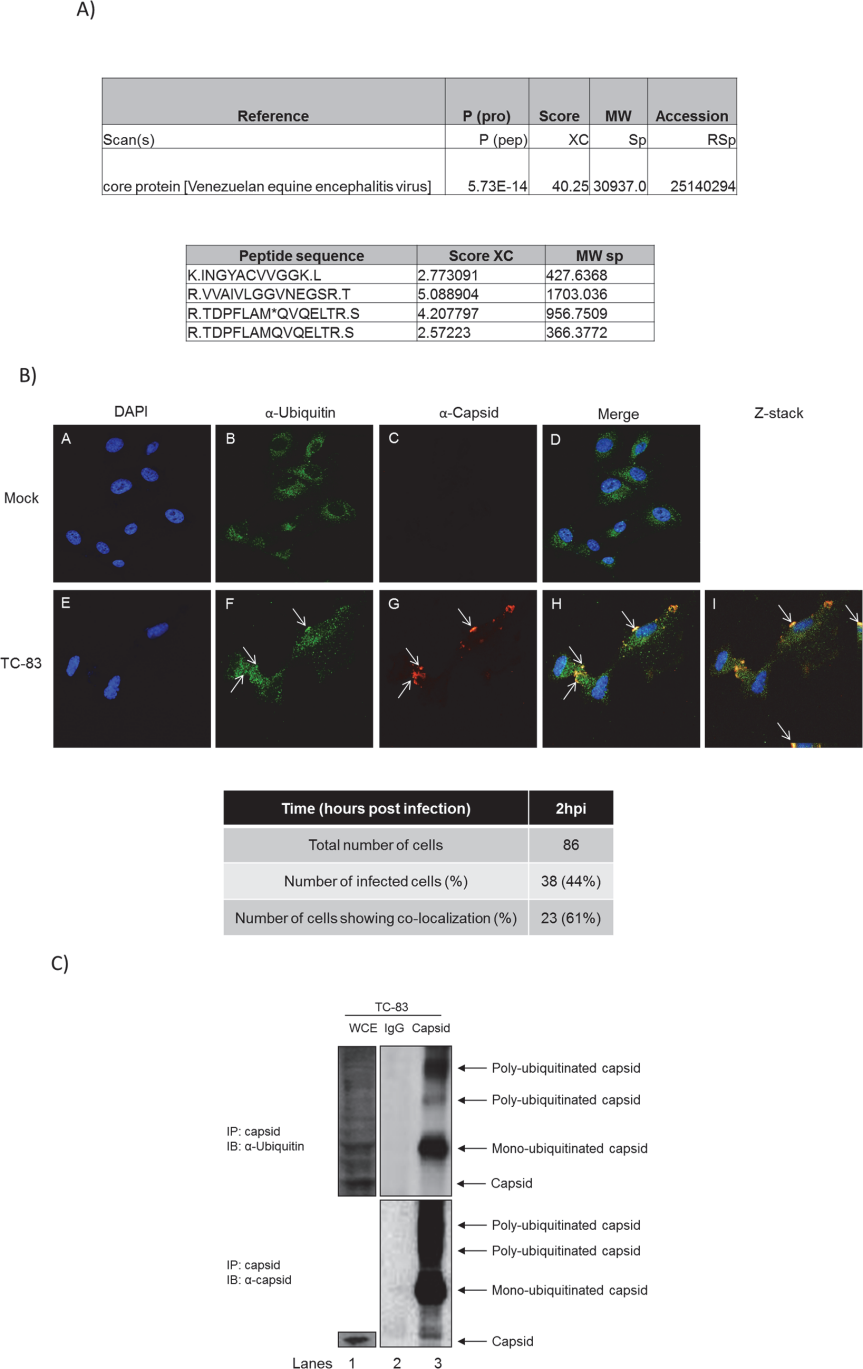
Ubiquitination of a viral protein. A) U87MG cells were transfected with HA-Ub for 24 hours and then infected with TC-83 (MOI: 5) for 1 hour. At 6 hours post infection, cell lysates were collected and quantified for protein concentration. Two milligrams of total protein was immunoprecipitated with HA probe antibody and mouse IgG as a control. LC/MS-MS was performed and the results tabulated. B) U87MG cells were seeded in an 8-well chambered slide at 20,000 cells per well. The cells were uninfected (Mock) or infected with TC-83 at an MOI of 10. At 2 hours post transfection, cells were fixed and processed as described in the materials and methods section. The cells were probed with ubiquitin and capsid antibodies followed by incubation with Alexa-Fluor 488 and Alexa-Fluor 568 respectively. The cells were stained with DAPI to observe the nuclei. Images were taken using Nikon Eclipse TE2000-U with a 60X objective and are representative of 2 independent experiments performed in duplicate. C) U87MG cells were infected with TC-83 (MOI: 5) for 1 hour. At 6 hours post infection, cell lysates were collected and quantified for protein concentration. Two milligrams of total protein was immunoprecipitated with ubiquitin antibody and an isotype IgG as a control. Immunoprecipitated samples were resolved by SDS-PAGE and subsequently immunoblotted for capsid (top panel) and ubiquitin (bottom panel). The image is representative of 3 independent experiments.

Our data thus far supports the idea that in Bortezomib-treated U87MG cells, VEEV replication decreased at early time points post infection. Hence, we hypothesized that the observed reduction was due to the inhibition of a viral protein(s) such as capsid that would normally be post translationally modified with ubiquitin which will ultimately have an effect on the intracellular viral RNA levels directly or indirectly. To address this hypothesis, U87MG cells were transfected with HA-Ub for 24 hours and then infected with TC-83. Cells lysates were prepared at 6 hours post infection. HA-Ub expression was validated by Western blot (data not shown). Total cell lysates were immunoprecipitated with HA antibody and an LC-MS/MS performed. Control IPs included an isotype IgG antibody. LC-MS/MS results were analyzed and tabulated in Fig 7A. Mass spectrometry analysis indicated that viral capsid associated with HA tagged ubiquitin in infected cells (Fig 7A). An in-depth investigation of the ubiquitination status of host and additional viral proteins during the entire time course of alphavirus infection and the impact on viral multiplication is ongoing in our lab. Confocal microscopy was performed to independently validate capsid ubiquitination using specific antibodies to detect capsid and ubiquitin. U87MG cells were infected with TC-83 and at 2 hours post infection the cells were fixed. This time point was selected as Bortezomib effectively inhibited TC-83 multiplication as early as 2 hours post infection (Fig 3). A total of 86 cells were counted of which 38 cells were infected. Co-localization (shown by the arrows in Fig 7B) of capsid with ubiquitin was observed in 23 of the infected cells. This translates to ~61% of infected cells displaying co-localization. Panels E–H (Fig 7B) serve as examples of infected cells in a given field of view. Panel I is a Z-stack image of panels E-H, where co-localization of capsid with ubiquitin is shown by the arrows. The co-localization was confirmed by Z-stack analysis, quantified and tabulated below Fig 7B. Immunoprecipitation analysis was performed to provide additional independent validation of the ubiquitination status of the capsid protein. Briefly, U87MG cells were infected with TC-83 and at 6 hours post infection cell lysates were prepared for immunoprecipitation. Cell lysates were incubated with a capsid antibody and an isotype IgG control antibody. The cell extracts were resolved by SDS-PAGE and the subsequent immunoblot was probed for ubiquitin and capsid (Fig 7C). At 6 hours post infection mono-ubiquitinated and poly-ubiquitinated capsid species were observed. Taken together these results indicated that VEEV capsid was ubiquitinated in the infected cells.

### TC-83 capsid was ubiquitinated on K48

**Fig 8:**
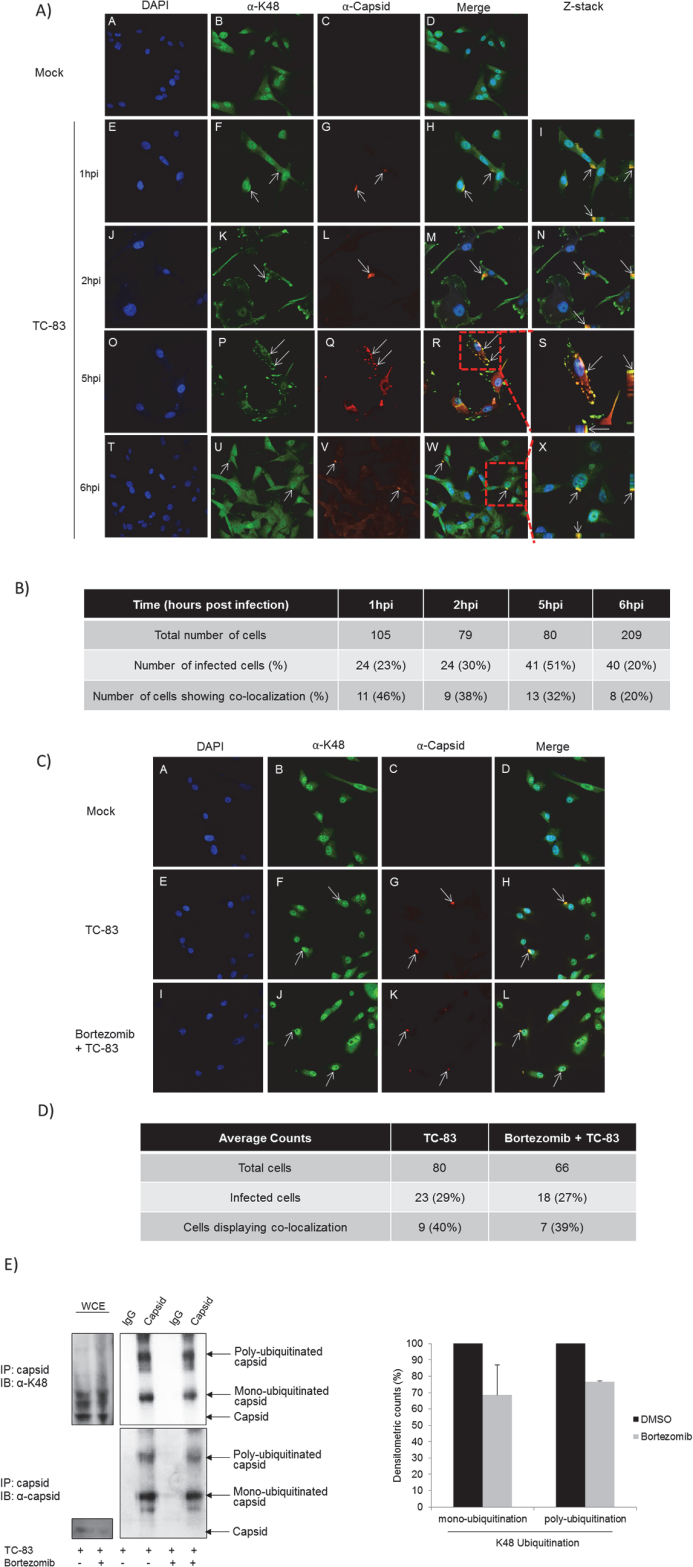
VEEV capsid protein is ubiquitinated on K48. A) U87MG cells were seeded in an 8-well chambered slide at 20,000 cells per well. The cells were uninfected (Mock) or infected with TC-83 at an MOI of 10. At 1, 2, 5 and 6 hours post infection, cells were fixed and processed as described in the materials and methods section. The cells were probed with K48 ubiquitin and capsid antibodies followed by incubation with Alexa-Fluor 488 and Alexa-Fluor 568 respectively. The cells were stained with DAPI to observe the nuclei. Images were taken using Nikon Eclipse TE2000-U with a 60X objective and are representative of 2 replicates in an experiment. Red boxes are parts of the image that have been zoomed in and displayed in Z-stacks. B) Number of infected U87MG cells showing co-localization tabulated. C) U87MG cells were seeded in an 8-well chambered slide at 20,000 cells per well. The cells were untreated (Mock), DMSO treated or Bortezomib treated (0.1μM) for 2 hours. The cells were uninfected (Mock) or infected with TC-83 at an MOI of 10. At 2 hours post infection, cells were fixed and processed as described in the materials and methods section. The cells were probed with K48 ubiquitin and capsid antibodies followed by incubation with Alexa-Fluor 488 and Alexa-Fluor 568 respectively. The cells were stained with DAPI to observe the nuclei. Images were taken using Nikon Eclipse TE2000-U with a 60X objective and are representative of 2 independent experiments performed in duplicate. D) Number of infected U87MG cells showing co-localization tabulated. E) U87MG cells were treated with Bortezomib (0.1μM) or DMSO for 2 hours and then infected with TC-83 (MOI: 5) for 1 hour. At 2 hours post infection cell lysates were collected, lysed and quantified. Equal amounts of total protein were immunoprecipitated with capsid antibody and resolved by SDS-PAGE and subsequently immunoblotted for K48 ubiquitin (top panel) and capsid (bottom panel). The image is representative of 2 independent experiments. Protein bands were quantified using Image J software and normalized to capsid. Average percent differences of 2 independent experiments are depicted graphically.

Collectively, the results thus far suggest that capsid is ubiquitinated in the early stages of infection which supports the requirement of a functional proteasome system which may permit degradation of the capsid and release of viral genomic RNA into the cytoplasm of infected cells. There is a potential for host proteins/factors that are ubiquitinated or that are prevented from degradation during alphavirus infections. In this manuscript however, we are focused on the capsid protein ubiquitination in an infected host cell. As K48 ubiquitination is known to target proteins for proteasomal degradation [37], therefore we determined whether TC-83 capsid is ubiquitinated through K48 linkage during the infectious process. To perform these studies, we used an antibody to specifically detect K48 ubiquitination in TC-83 infected cells using confocal microscopy. U87MG cells were infected with TC-83 and at 1, 2, 5 and 6 hours post infection, cells were fixed and probed for K48 ubiquitin and capsid. Co-localization of capsid with K48 ubiquitin was observed at all-time points investigated, as shown by the arrows in Fig 8A. Panels E–X in Fig 8A serve as examples of infected cells in a given field of view. Panels I and N are Z-stack images of panels H and M respectively. Panels S and X are zoomed in Z-stack images of panels R and W respectively (as referenced by the red boxes). Co-localization of capsid with K48 ubiquitin is shown by the arrows in Fig 8A. The co-localization was confirmed by Z-stack analysis, quantified and tabulated in Fig 8B. As early as 1 hour post infection, 46% co-localization of capsid and K48 ubiquitin was observed. At 2, 5 and 6 hours post infection co-localization was recorded to be 38%, 32% and 20%, respectively. Confocal microscopy with α-K63 ubiquitin did not reveal a clearly discernable signal with the capsid protein (data not shown). These data indicate that during the course of infection K48 ubiquitination of capsid decreased. Since proteasomal inhibitors act to deplete free ubiquitin [3], we hypothesized that Bortezomib pre-treatment will decrease the available pool of free ubiquitin in the system resulting in a decrease in K48 ubiquitination of capsid when compared to the untreated cells. This was addressed by confocal microscopy, whereby U87MG cells were pre-treated with DMSO, Bortezomib or untreated (Mock) and either infected or uninfected with TC-83. At 2 hours post infection, cells were fixed and probed for capsid and K48 ubiquitin. Co-localization of capsid with K48 ubiquitin (40%) was observed with the TC-83 infected cells, as shown by the arrows in Fig 8C (Panels E-H). This outcome was comparable to the result in Fig 8B (compare Fig 8B (38%) with Fig 8D (40%)). K48 ubiquitination (39%) was also observed in the Bortezomib treated infected cells (Panels I-L).Panels E–L in Fig 8C serve as examples of infected cells in a given field of view. The co-localization was confirmed by Z-stack analysis, quantified and tabulated in Fig 8D. Since the K48 ubiquitin confocal microscopy assay did not distinguish between mono-ubiquitination and poly-ubiquitination species of capsid a co-immunoprecipitation was performed. U87MG cells were pre-treated with Bortezomib or DMSO and then infected with TC-83. Cells were collected at 2 hours post infection lysed and quantified. Equal amounts of total protein were immunoprecipitated with capsid and an isotype IgG control. The subsequent immunoblot was probed for K48 ubiquitination (Fig 8E). We observed a decrease in the band intensities of both mono-ubiquitinated and poly-ubiquitinated capsid with Bortezomib treatment. Densitometric counts represented graphically illustrated a 31% decrease in K48 mono-ubiquitination and a 23% decrease in K48 poly-ubiquitination with Bortezomib treatment when compared to the DMSO controls. Thus far, these results indicated that at early time points of infection capsid was K48 ubiquitinated which was affected by Bortezomib treatment.

### Capsid protein in TC-83 virions was ubiquitinated

**Fig 9:**
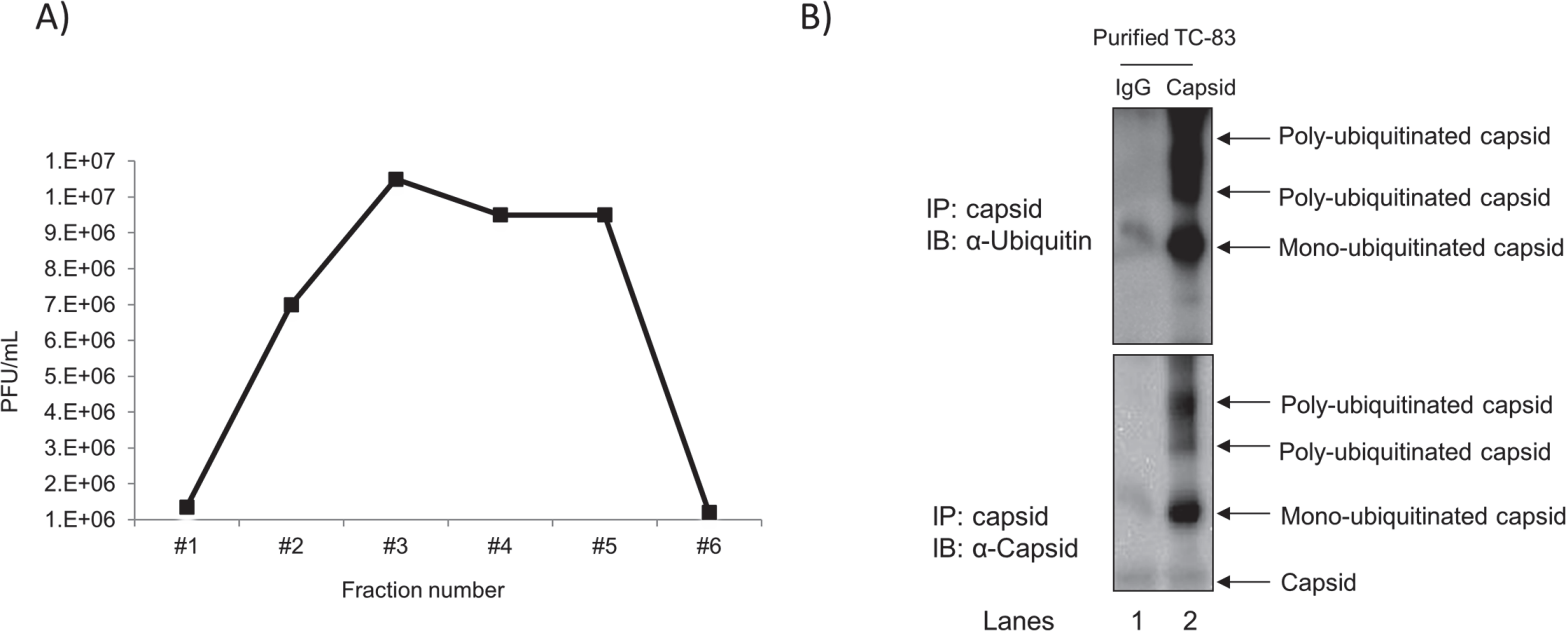
Ubiquitinated capsid in virions. VEEV-containing supernatants obtained from infected VERO cells were subjected to sucrose density centrifugation as described in the materials and methods section. A) The collected fractions were analyzed by plaque assay and are represented graphically. Fraction 3 with the highest titer of virus was used for immunoprecipitation with capsid antibody and an isotype IgG as a control. Immunoprecipitated samples were resolved by SDS-PAGE and subsequently immunoblotted for ubiquitin (top panel) and capsid (bottom panel). The image is representative of duplicate immunoprecipitation runs.

We have shown that Bortezomib inhibited TC-83 at early stages of infection (Figs 3 and 6), which supports a probable mechanism that Bortezomib may inhibit ubiquitin mediated degradation of capsid to allow release of viral genomic RNA into the cytoplasm of infected cells. This contributes to the idea that the incoming virions should be ubiquitinated on the capsid protein; hence a relevant population to analyze would be the virions themselves. We attempted to determine whether the capsid protein in the virions was ubiquitinated. To address this, we performed in triplicate a sucrose density centrifugation of virus-enriched supernatants obtained from infected VERO cells. Six fractions were analyzed by plaque assay to determine the fraction with the purest viral suspension (Fig 9A). Fraction 3 depicting the highest titer of sucrose purified virus (1E7 PFU/mL) was chosen for immunoprecipitation with capsid antibody and an isotype IgG antibody as a control. The subsequent blot was probed for ubiquitin and capsid and is shown in Fig 9B. This observation was validated in independent duplicate immunoprecipitation runs. Mono-ubiquitinated and poly-ubiquitinated forms of capsid were observed which added evidence to the idea that capsid protein exists in an ubiquinated form in VEEV virions.

## Discussion

VEEV is an emerging infectious virus that causes natural outbreaks in many parts of the world [38]. VEEV was weaponized in the past and continues to be classified as a bio-threat agent owing to the retention of stability and infectivity in the aerosol form [21]. The diverse roles that the UPS plays in a cell make it an attractive target for pathogens, particularly viruses. The UPS is involved in protein degradation, protein trafficking, transcription and cell signaling, hence viruses can easily manipulate this system to enhance replication [39]. Recently, many viruses have been demonstrated to be involved in modifying the UPS to enhance replication, such as Influenza virus and mouse hepatitis virus [3,7,8]. In this study, we aimed to investigate the effects of a functional host proteasome system on alphavirus replication.

The FDA approved drug, Bortezomib (Velcade), was used as a proteasomal inhibitor to demonstrate broad spectrum efficacy against the attenuated TC-83 strain and the virulent strains of VEEV, EEEV and WEEV at non-toxic concentrations (Figs 1, 2, 3 and 5). Also, Bortezomib treatment indicated that a functional UPS at early stages of the viral life cycle is necessary for efficient viral multiplication (Fig 3). The inhibition observed with Bortezomib treatment was not drug specific as a similar inhibitory trend was observed with MG132 (Fig 4). The greater extent of viral inhibition observed with MG132 treatment was likely attributed to the inhibition of additional targets of MG132, such as certain lysosomal cysteine proteases, calpains and cathepsins [40,41]. For example, a study by Schneider et al., highlighted that MG132 inhibition of SARS-CoV replication was due to m-calpain inhibition—a proteasomal independent mechanism of inhibition [4]. This could explain the more effective inhibition of MG132 over Bortezomib that was observed; Bortezomib, on the other hand, is a highly specific inhibitor of the proteasome with no additional off target effects and is also documented to be reversible. One important concern with the potential application of Bortezomib as an antiviral therapeutic may be that the proteasome plays an integral role in the life of a cell and inhibiting the proteasome may have deleterious consequences to the host; however, the fact that Bortezomib-mediated inhibition of the proteasome is a reversible process and that the treatment regimen is likely to be restricted to short time windows can justify the utility of this inhibitor as a therapeutic. Additionally, by inhibiting the proteasome function, inhibition of inflammation can also be achieved (by interfering with the degradation of IκBα and hence, nuclear translocation of p65 and transcriptional activation) which can have important consequences to neuronal encephalitis.

Collectively, these observations dove tails with the reported evidences in the literature that Bortezomib is an inhibitor of multiple viruses, including Nipah virus and mouse hepatitis coronavirus [11,42,43]. Bortezomib inhibited mouse hepatitis coronavirus replication early in the infectious process, and the inhibition was likely due to the inhibition of viral entry and/or viral RNA synthesis [11,43]. Comparatively, in our study, Bortezomib treatment decreased intracellular VEEV genomic copies and modulated RNA assembly into relevant cytoplasmic structures (Fig 6). It is important to note that the particular aspects of the viral infectious cycle that are influenced by Bortezomib however, is different for the various viruses thus suggesting that different viral families may utilize the host UPS for ubiquitin mediated degradation and/or signaling events that facilitate the establishment of a productive viral infection. Such differences in ubiquitination of viral targets may also be dependent on tropism and the nature of the host cell type that is used in these experiments.

Horan et al., recently demonstrated that HSV-1 capsid was K48 ubiquitinated in the cytoplasm of infected macrophages and degraded by the proteasome for release of genomic DNA for detection by host DNA sensors to induce an antiviral response [13]. Here, we validated by confocal microscopy (Fig 7B) and immunoprecipitation (Fig 7C) that capsid was ubiquitinated during the course of infection. In addition, we have shown that VEEV capsid was K48 ubiquitinated early in infection (Fig 8A), which may allow for release of the viral RNA for efficient RNA translation and replication. Interestingly, only 48% co-localization of capsid with K48 ubiquitin was observed, whereas 61% co-localization of capsid with total ubiquitin was observed at the same time point. However, over time a modest decline in K48 ubiquitination of capsid was recorded. This could be ascribed to 3 possible reasons. Firstly, not all of the incoming capsid may be K48 ubiquitinated as there could be other forms of ubiquitination, such as K63, K11 and so forth. Secondly, since capsid is a multi-functional viral protein, not all of the capsid within a cell will be destined for a single function at a time. Thirdly, in an *in vitro* cell culture system there may be variability and differences in infectivity and signaling patterns which may also be attributed to an unsynchronized cell population. Moreover, the capsid in virions appeared to be ubiquitinated (Fig 9). This observation, may share a similarity to a Nipah virus study conducted by Wang et al.; where the UPS was required for nuclear cytoplasmic trafficking of the matrix protein, which was necessary for viral budding [42]. Ongoing studies in our laboratory are focused on elucidating the type of ubiquitination that occurs on capsid in virions, and if ubiquitination of the capsid protein is required for viral egress.

In conclusion we have shown that VEEV requires a functional UPS for efficient replication. During early stages of infection, VEEV capsid is ubiquitinated and most likely requires a functional proteasome system for degradation to allow viral RNA to be released into the cytoplasm for RNA translation and replication to occur. Additional in-depth studies such as pulse chase experiments using radiolabels may shed light on the actual stabilization of capsid in the presence of proteasomal inhibitors. Further studies are needed to elucidate if other viral proteins require ubiquitination to ensure translation and synthesis of VEEV RNA and such investigation are ongoing in our laboratory. Such future work will aid our understanding of alphavirus biology and viral manipulation of the UPS system, which will pave the way for novel therapeutic strategies.
